# Ascorbate-catalyzed reductive nitrosylation of ferric myoglobin

**DOI:** 10.1016/j.rbc.2026.100088

**Published:** 2026-08-17

**Authors:** Thilini Karunarathna, Stephen R. Baker, Julian Flanagan, Troy A. Stich, Jesús Tejero, Matthew R. Dent, Elizabeth Rochon, Anthony W. DeMartino, Mark T. Gladwin, Daniel B. Kim-Shapiro

**Affiliations:** a Department of Physics, Winston-Salem, NC, 27109, USA; b Translational Science Center, Winston-Salem, NC, 27109, USA; c Department of Chemistry Wake Forest University, Winston-Salem, NC, 27109, USA; d Heart, Lung, Blood, and Vascular Medicine Institute, University of Pittsburgh, Pittsburgh, PA, 15261, USA; e Division of Pulmonary, Allergy and Critical Care Medicine, University of Pittsburgh, Pittsburgh, PA, 15261, USA; f Department of Bioengineering, University of Pittsburgh, Pittsburgh, PA, 15260, USA; g Department of Pharmacology and Chemical Biology, University of Pittsburgh, Pittsburgh, PA, 15261, USA; h Department of Chemistry, Wayne State University, Detroit, MI, 48202, USA; i Department of Medicine, University of Maryland School of Medicine, Baltimore, MD, 21201, USA

**Keywords:** Nitric oxide, Myoglobin, Ascorbic acid, Glutathione, Lipid peroxidation, Spectroscopy

## Abstract

Myoglobin plays a key role in oxygen storage and delivery through oxygen binding and release from the ferrous (Fe^+2^) heme. However, when the heme is oxidized to its ferric (Fe^+3^) form it can no longer bind oxygen and further oxidation leads to lipid peroxidation which can lead to a variety of pathological consequences. Ferric heme can be reduced to the ferrous form by nitric oxide (NO^•^) through classical reductive nitrosylation but this reaction, where a second NO^•^ molecule binds to the reduced heme to form iron nitrosyl myoglobin, is very slow and inefficient. We have recently demonstrated that glutathione (and other thiols) can catalyze reductive nitrosylation of free ferric heme (hemin) when solubilized in albumin or red blood cell ghosts and that the resultant NO-ferroheme has potent NO^•^ signaling properties. In this work we show this catalysis is also viable in myoglobin and that ascorbate is a better catalyst than glutathione with reductive nitrosylation occurring 1000 times faster in the presence of ascorbate compared to its absence under some conditions. Our data support a mechanism involving ascorbate-mediated heme reduction forming an ascorbyl radical. Only one NO^•^ molecule and 0.5 ascorbate molecules are needed to convert one ferric myoglobin to one nitrosyl myoglobin molecule. Addition of NO^•^ and ascorbate are shown to be more protective against lipid peroxidation due to myoglobin oxidation by hydrogen peroxide than either ascorbate or NO^•^ alone. This reaction has potential application in meat preservation and treatment of ischemic reperfusion injury, various myopathies, and rhabdomyolysis.

## 1. Introduction

The primary function of myoglobin (Mb) is to store oxygen that can be used in the surrounding tissue. Their heme groups bind oxygen when the iron is in the ferrous (+2) oxidation state. When the iron is oxidized to the ferric oxidation state (+3), the heme cannot bind oxygen. Moreover, the ferric heme can facilitate oxidative damage such as lipid peroxidation, particularly if it is further oxidized to the ferryl oxidation state (+4) and (when oxidized by hydrogen peroxide) an associated protein free radical [1–4],

(1) $$\text{Mb}\left({\text{Fe}}^{+3}\right)+{\text{H}}_{2}{\text{O}}_{2}\to {\text{Mb}}^{•}\left({\text{Fe}}^{+4}\right)=0+{\text{H}}_{2}\text{O}.$$

In addition, heme-globins lose their hemes more easily when they are in the ferric state and free heme is a danger-associated molecular pattern (DAMP) [5]. Thus, cells employ mechanisms to maintain the heme in the ferrous state [6].

In addition to storing oxygen, Mb is largely involved in nitric oxide signaling [7–11]. The association rate constant for NO^•^ and ferrous heme Mb is on the order of 10^7^ M^−1^s^−1^ [12]. The association rate constants is smaller when the heme is oxidized (Fe^+3^, as low as 10^3^ M^−1^s^−1^) [12] but the lower overall affinity of the ferric hemes for NO^•^ compared to ferrous hemes in Mb is mainly due to its relatively fast dissociation rate constants (1–40 s^−1^ vs 10^−3^−10^−5^ s^−1^) [12]. Thus, ferric heme nitrosyl species are not very stable (with the NO^•^ coming off relatively rapidly), while the ferrous heme nitrosyl is stable and has recently been explored as a potent signaling agent, NO-ferroheme [9,11]. Ferric nitrosyl species can be converted to ferrous nitrosyl ones through reductive nitrosylation [10]. In the case of Mb,

(2) $$\text{Mb}\left({\text{Fe}}^{+3}-\text{NO}\right)+{\text{OH}}^{-}\to \text{Mb}\left({\text{Fe}}^{+2}\right)+{\text{HNO}}_{2}.$$

Following reduction to the ferrous form, another NO^•^ molecule (if available) quickly binds so that two NO^•^ molecules are required to form one ferrous iron nitrosyl Mb.

The kinetics of this reaction is governed by rate constants of the form [10].

(3) $$k_{\text{Mb}}=k_{\text{OH}}\prime \frac{K[{\text{NO}}^{\cdot }]}{1+K[{\text{NO}}^{\cdot }]}$$

where *k*_Mb_ refers to the first order rate constant for formation of ferrous nitrosyl Mb, *K* is the equilibrium constant for NO^•^ binding to ferric heme, and

(4) $$k_{\text{OH}}’=k_{\text{OH}}\left[{\text{OH}}^{-}\right],$$

where *k*_OH_ is the second order rate constant for the reaction of ferric nitrosyl Mb and hydroxyl ion [10]. The maximum rate constants at neutral pH occur at high NO^•^ concentrations where the product *K*[NO^•^] >1which would be greater than 100 μM NO^•^ [10,12]. The observed rate constants at neutral pH (based on previous measurements of *k*_OH_) [10] are then predicted to be 3 × 10^−5^ s^−1^, with a corresponding half-life of about 380 min. Given the relatively fast dissociation rate constants quoted above, ferric heme protein reduction by NO^•^ via equation (2) would be very inefficient at neutral pH. Moreover, since formation of NO-ferroheme would subsequently require a second NO^•^ to bind the ferrous heme, classical reductive nitrosylation of ferric myoglobin is predicted to be nearly impossible under physiological or therapeutic conditions.

Although classical reductive nitrosylation is predicted to be highly inefficient under physiological conditions, thiol catalyzed reductive nitrosylation that we have recently discovered may be viable [9]. The reaction, with glutathione (GSH) as the catalyst proceeds as

(5) $${\text{Fe}}^{+3}-\text{NO}+\text{GSH}\to {\text{Fe}}^{+2}-\text{NO}+{\text{GS}}^{•}+{\text{H}}^{+},$$

where the thiyl radical could form a nitrosothiol by reacting with another NO^•^ if available or undergo other reactions leading to disulfide formation or other species [9]. In the presence of GSH, the reaction was observed to be governed by a rate constant of up to 10^4^ M^−1^ s^−1^ at neutral pH [9]. The reaction was demonstrated with heme solubilized in albumin, red blood cell membrane ghosts, and a methanol/aqueous buffer system [9]. We have subsequently shown that a similar reaction is catalyzed by sulfide [13]. The relatively fast kinetics, combined with the fact that only one NO^•^ molecule is required to make one NO-ferroheme suggests that these reactions may be viable under physiological or therapeutic conditions.

NO-ferroheme formation could occur through an inner sphere or outer sphere mechanism [9]. The inner sphere mechanism involves GSH binding to the heme distal to NO^•^ so that a transient hexacoordinate heme is formed with both NO^•^ and GSH bound and GSH transfers an electron. In the outer sphere mechanism, GSH is in proximity to the heme but not covalently bound. In this paper, we report that ascorbate catalyzes reductive nitrosylation in Mb where the proximal histidine precludes an inner sphere mechanism.

## 2. Materials and methods

### 2.1. Materials

Unless otherwise indicated, all materials were obtained from Sigma-Aldrich. Stock solutions of phosphate-buffered saline (PBS) and 20 mM sodium hydroxide were prepared in glass vials and sparged with argon gas for 1 h prior to use. Metmyoglobin (bovine (Worthington Biomedical Corporation), equine (Sigma-Aldrich), and recombinant human), ascorbate (Sigma-Aldrich), GSH (Alexis Biochemicals), DEA NONOate, and PROLI NONOate (Caymen Chemical Company) stocks were prepared by degassing each compound with argon before adding the respective solvents. The concentrations of metmyoglobin stocks were verified by absorption spectroscopy using extinction coefficients at 409 nm: 157 mM^−1^ cm^−1^ for bovine [14], 188 mM^−1^ cm^−1^ for equine [15], and 179 mM^−1^ cm^−1^ for human [16] metmyoglobin. Unless otherwise specified, bovine metmyoglobin was used for all experiments. The stock concentration of PROLI NONOate was confirmed by absorption spectroscopy, using an extinction coefficient of 8.4 mM^−1^ cm^−1^ at 248 nm [9]; similarly, the ascorbate stock concentration was verified by absorption spectroscopy using an extinction coefficient of 13 mM^−1^ cm^−1^ at 265 nm [17]. The concentration of NO^•^ released from PROLI NONOate was quantified by titrating 25 μM PROLI NONOate with 50 μM oxyhemoglobin, followed by deconvolution of the resulting methemoglobin/oxyhemoglobin spectrum by fitting to normalized basis spectra. We measured that one PROLI NONOate produced 1–1.2 NO^•^ molecules. We measured the half-life of PROLI NONOate by quickly mixing it with excess oxyhemoglobin and monitoring the absorbance vs time at 630 nm. We found the half-life to be 4.9 ± 0.9 s in PBS buffer at room temperature, consistent with the reported PROLI NONOate half-life value of 6.4 s at 22 °C, pH 7.4 [18]. For DEA NONOate, we found that one DEA NONOate molecule released 1.8 ± 0.4 NO^•^ molecules by following methemoglobin formation after DEANONOate was added to oxyhemoglobin and we found the half-life of DEANONOate was 13 ± 2 min (similar to that reported previously (16 min) at 22 °C, pH 7.4 [19]).

Recombinant human myoglobin was produced in *Escherichia coli* as a GADPH-Mb fusion protein. The expression plasmid was constructed by Genscript (Piscataway, NJ) as follows. A DNA fragment was synthesized including the sequences of human GAPDH (Uniprot accession code P04406) and human myoglobin (Uniprot accession code P02144) joined by a four glycine spacer sequence. A 6xHistg was included in the C-terminus of the fragment. The DNA was inserted in the NcoI/HindIII sites of the pET28a plasmid (Novagen).

Expression and purification was performed by similar procedures to those used for cytoglobin [20]. The bacterial cultures were supplemented with δ-aminolevulenic acid (0.4 mM) to promote heme synthesis and induction was performed with 1 mM isopropyl β-d-1-thiogalactopyranoside. The GAPDH-Mb containing a C-terminal Histag was purified by Ni-nitrilotriacetic acid (Ni-NTA) agarose chromatography using an Akta Purifier 10 FPLC system (GE Healthcare) with UNICORN software as described [20]).

### 2.2. Reductive nitrosylation kinetics

Reductive nitrosylation kinetics were monitored using UV-vis absorption spectroscopy at room temperature. In brief, all reactions were observed from 450 to 700 nm for 1–6 h during each step of the reaction. The classical reductive nitrosylation reaction was observed by combining 50 μM metmyoglobin with 200 μM NO^•^ and monitoring it for 6 h. The catalyzed reductive nitrosylation was conducted using 50 μM metmyoglobin and 200 μM NO^•^ in the presence of 2 mM GSH or 2 mM ascorbate for 1 h. All reactions were carried out under both anaerobic and aerobic conditions. Other reactant concentrations used are given in the text. All reactions were performed at room temperature unless otherwise noted.

Kinetics were analyzed analogously to that described previously [10] and described in Equations (3) and (4). We have

(6) $$\frac{\text{d}\left[{\text{Mb}}^{+2}-\text{NO}\right]}{\text{dt}}=-k_{\text{Asc}}[\text{Asc}]\left[{\text{Mb}}^{+3}-\text{NO}\right]$$

where *k*_Asc_ is the bimolecular rate constant governing the reductive nitrosylation. We can generalize Equation (4) by rewriting Mb^+3^ – NO].

(7) $$\left[{\text{Mb}}^{+3}-\text{NO}\right]=\frac{\text{K}[{\text{NO}}^{\cdot }]}{1+\text{K}\left[{\text{NO}}^{\cdot }\right]}\left(\left[{\text{Mb}}_{\text{tot}}\right]-\left[{\text{Mb}}^{+2}-\text{NO}\right]\right)$$

As the total amount of Mb ([Mb_tot_]) is constant, when [NO] is sufficiently in excess to [Mb], reductive nitrosylation is governed by first order kinetics with an observed rate constant of

(8) $$k_{\text{Mb}}=\frac{\text{K}[{\text{NO}}^{\cdot }]}{1+\text{K}[{\text{NO}}^{\cdot }]}k_{Asc}[\text{Asc}]$$

which is comparable to Equation (4) and where *K* is the equilibrium constant for NO^•^ binding as defined above. The equilibrium constant was calculated by titrations of NO with Mb and obtaining the fraction that is bound to NO^•^ by deconvoluting with basis spectra. Kinetics for GSH are analyzed identically with *k*_*Asc*_ being replaced by *k*_*GSH*_.

### 2.3. Electron paramagnetic spectroscopy

Room temperature electron paramagnetic spectroscopy (EPR) was performed to confirm the presence of the ascorbyl radical using an Bruker EMX spectrometer operating at 9.4 GHz, 1G modulation, 10 mW power, and 100 ms time constant. A stock solution of 200 μM ascorbate was made in pH 7.4 PBS and added to quartz capillary tubes, which were placed into quartz EPR tubes prior to scanning. To confirm the presence of the ascorbyl radical in the stock solution, the magnetic field was scanned from 3334 to 3352 G to detect the distinct EPR doublet signal. To confirm the presence of the ascorbyl radical in our reaction, 50 μM Mb, 200 μM ascorbate, and 50 μM NO^•^ were mixed, quickly added to a glass capillary tube, and placed into a glass EPR tube prior to scanning. The reaction was then monitored, scanning over 3334–3352 G every 20 s for 1 h. EasySpin (EasySpin.org) was used to analyze the data. The signal amplitude was quantified by performing a double integral of the signal.

Low temperature EPR was performed on a Bruker EMX spectrometer as described previously [9]. The microwave frequency was set to 9.4 GHz and 5 G modulation, 10 mW power, 328 ms time constant, and 164 s scan over 600 G at 110 K were employed.

### 2.4. Determination of dehydroascorbic acid (DHA) formation

The formation of DHA was measured by loss of absorbance due to ascorbate. Ascorbate concentration was measured using absorption spectroscopy based on an extinction coefficient of 13 mM^−1^ cm^−1^ at 265 nm [17]. To verify that the loss of ascorbate was due to DHA formation, we added dithiothreitol (DTT) which reduces the DHA back to ascorbate. In mixtures containing myoglobin, the ascorbate and DHA were separated from the myoglobin using Centricon filters (10 KD MW cutoff).

### 2.5. Lipid peroxidation

Lipid peroxidation was determined using a Thiobarbituric acid reactive substances (TBARS) assay (R&D systems, Inc.). In brief, 50 μM metmyoglobin was mixed with either 1 mM ascorbate,100 μM NO^•^, neither, or both, and mixed with 0.4 mM arachidonic acid. Hydrogen peroxide (0.5 mM) was subsequently added to all samples, which were then incubated at 37 °C for 10 min. All samples were then mixed 1:1 with TBARS acid reagent, incubated for 15 min, and centrifuged at 12,000 g for 4 min. The supernatant was then removed, placed into wells of a 96-well plate, and mixed with thiobarbituric acid (TBA). The plate was then incubated at 50 °C for 2 h and the absorbance was read for each well at 532 nm. All samples were compared to a standard curve to determine TBARS concentrations. Samples were run in duplicate for each experiment, and experiments were run n = 4 times.

## 3. Results

### 3.1. Ascorbate accelerates NO-ferroheme formation in metmyoglobin

**Fig. 1.**
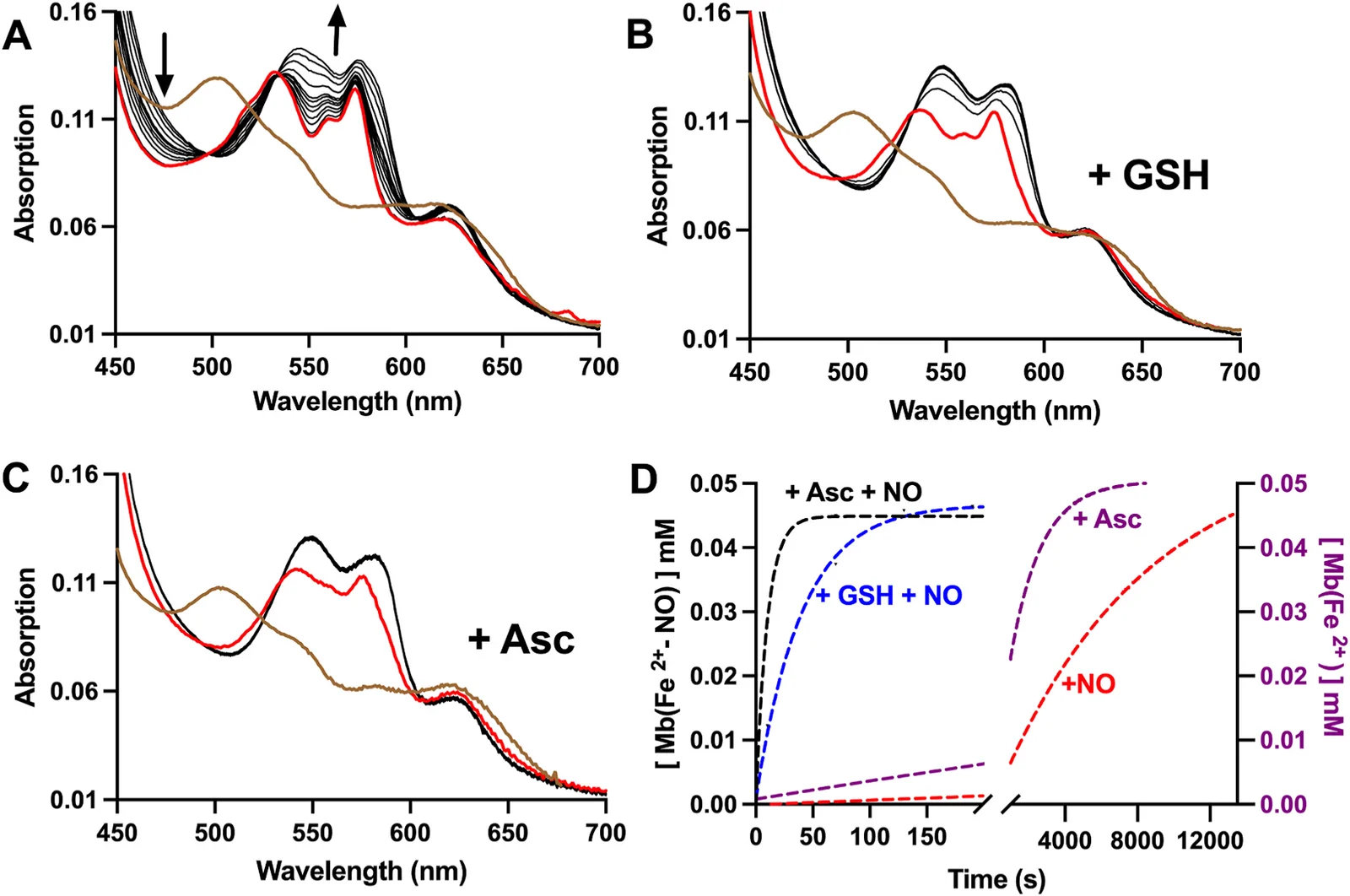
Kinetics of reductive nitrosylation. NO^⋅^ (200 μM) was added to metmyoglobin (50 μM) in the absence (A) or presence of (B) 2 mM GSH in Mops buffer (10 mM, pH 7.3) (C) 2 mM ascorbate in PBS buffer. Spectra of metmyoglobin prior to NO^⋅^ addition are shown (brown) as well as initial (red), intermediate, and final (black bold) spectra after NO^⋅^ addition. Spectra were recorded every 10 min for panel A and every 1 min for panels B and C. (D) Ferrous nitrosyl myoglobin content (and deoxygenated reduced myoglobin for the curve with ascorbate only) from spectral deconvolution vs time – note different time scales. All curves are plotted against the left axis except for ascorbate only (+Asc) which uses the right axis. Observed rate constants were 1.2 × 10^−4^ 1/s (+ NO^⋅^), 0.02 1/s (+GSH + NO^⋅^), 0.10 1/s (+Asc + NO^⋅^), and 5.8 × 10^−4^ 1/s (+Asc).

Our previous work demonstrated that GSH accelerates reductive nitrosylation of ferric heme to form NO-ferroheme with the heme being solubilized in albumin, red cell membrane ghosts, or a methanol/aqueous buffer mixture [9]. The presence of the proximal histidine bond in metMb could (in principle) preclude this thiol facilitated reductive nitrosylation but we found otherwise. As shown in Fig. 1A, metmyoglobin was mixed with NO^•^ under anaerobic conditions, producing NO-bound ferric myoglobin (red spectrum), which gradually converted to Mb(Fe^+2^-NO) through classical reductive nitrosylation. This occurred with an observed rate constant of 1.2 × 10^−4^ s^−1^, as evidenced by UV-Vis bands at 543 nm and 568 nm [9]. Adding 2 mM GSH, under similar conditions as in Fig. 1A, led to the rapid formation of NO-ferroheme, with a rate constant of 0.02 s^−1^ (Fig. 1B). Since ascorbate is another potential therapeutic agent that can reduce oxidative stress, we also examined NO-ferroheme formation in metmyoglobin in the presence of ascorbate. Ascorbate significantly accelerated Mb(Fe^+2^-NO) formation, resulting in a rate 1000 times faster than classical reductive nitrosylation (Fig. 1A vs 1C). Fig. 1D shows the temporal progress curves for classical reductive nitrosylation and that accelerated by GSH and ascorbate. The equilibrium constant for NO^•^ binding to ferric heme in metmyoglobin was determined to be (1.5 ± 0.1) × 10^−2^ μM^−1^ (n = 3), and the bimolecular rate constants in the presence of GSH (*k*_GSH_) and ascorbate (and *k*_Asc_) were determined to (16.0 ± 3.0) M^−1^ s^−1^ (n = 4) and (76.0 ± 9.0) M^−1^ s^−1^ (n = 7), respectively. When only ascorbate was added (without NO^•^), the rate constant for reduced deoxyMb formation was observed to be (2.1 ± 0.1 × 10^−1^) M^−1^s^−1^ (n = 4) demonstrating that Mb(Fe^+2^-NO) in the presence of ascorbate is not due to reduction of the ferric heme followed by NO^•^ binding.

### 3.2. Mb(Fe^+2^-NO) formation under oxygenated conditions

**Fig. 2.**
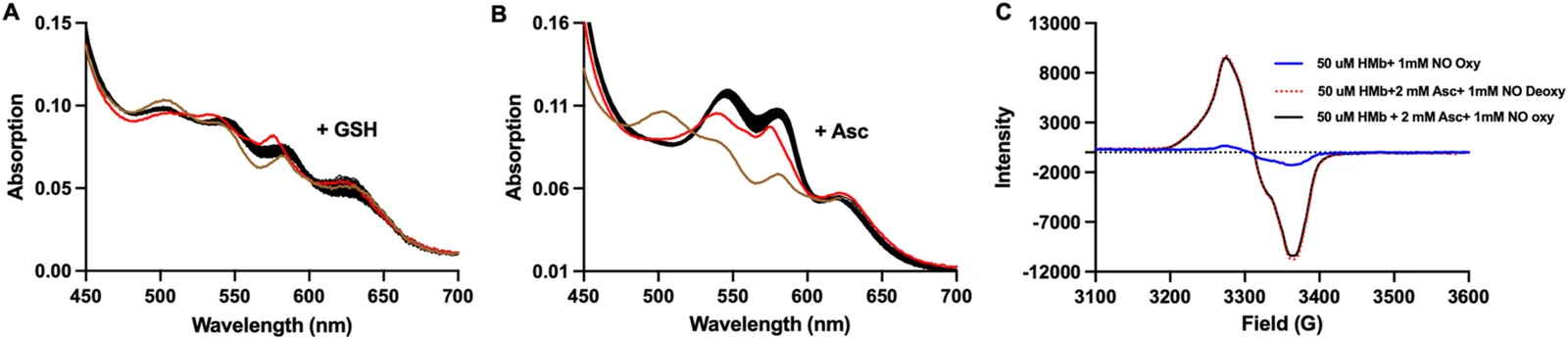
Kinetics of reductive nitrosylation under aerobic conditions. Absorption spectra when NO^⋅^ (200 μM) was added to metmyoglobin (50 μM) in the presence of (A) 2 mM GSH and (B) 2 mM ascorbate in PBS buffer. Spectra of metmyoglobin prior to NO^⋅^ addition are shown (brown) as well as initial (red), intermediate, and final (black bold) spectra after NO^⋅^ addition. The observed rate constant was 0.1 1/s with ascorbate. (C) Electron Paramagnetic Resonance. The lack of characteristic sharp hyperfine features supports the formation of hexacoordinate Mb(Fe^+2^-NO) when NO^⋅^ is mixed with equine metmyoglobin (50 μM) in the presence of ascorbate under aerobic and anaerobic conditions (overlapping spectra), but the reaction is relatively unproductive in the absence of ascorbate under aerobic conditions.

Given its role as a therapeutic agent and signaling molecule under physiological conditions, understanding Mb(Fe^+2^-NO) formation in aerobic environments is important. We performed experiments similar to those conducted under anaerobic conditions. The results indicate that ascorbate promotes Mb(Fe^+2^-NO) formation in both aerobic (Fig. 2B) and anaerobic (Fig. 1C) settings, with an observed rate constant of 0.1 s^−1^ under aerobic conditions. Conversely, GSH did not facilitate its formation (Fig. 2A). The EPR spectra (Fig. 2C), which lacked characteristic sharp hyperfine features, support the formation of hexacoordinate Mb(Fe^+2^-NO) when 1 mM NO^•^ was mixed with 50 μM equine metmyoglobin in the presence of ascorbate under both aerobic and anaerobic conditions (Fig. 2C, overlapping brown and dashed red spectra), whereas the reaction was relatively unproductive in the absence of ascorbate under aerobic conditions.

### 3.3. Mb(Fe^+2^-NO) formation across myoglobin species

**Fig. 3.**
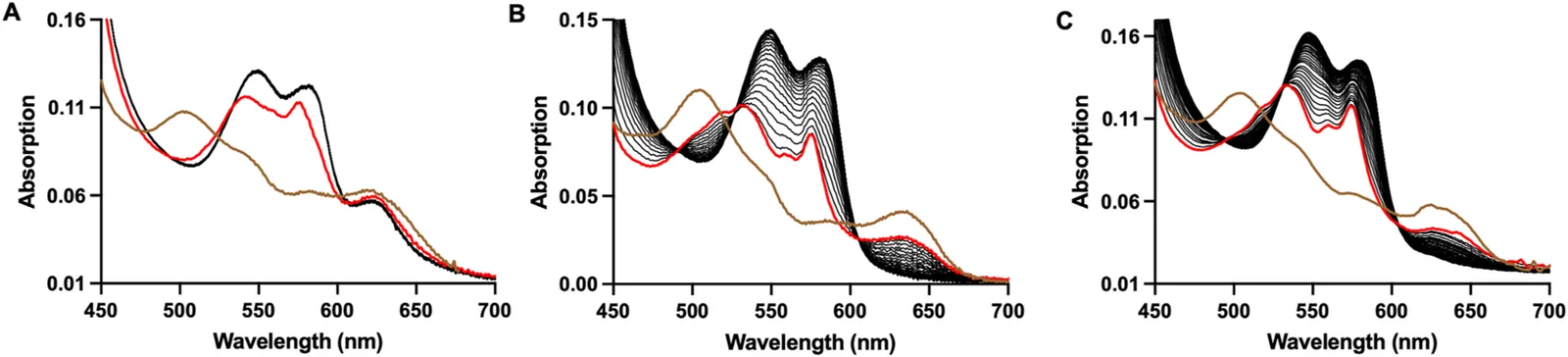
Kinetics of reductive nitrosylation in different species. NO^⋅^ (200 μM) was added to the 50 μM (A) Bovine (B) equine (C) Human metmyoglobin in the presence of 2 mM ascorbate in PBS buffer. Spectra of metmyoglobin before NO^⋅^ addition are shown (brown) as well as initial (red), intermediate, and final (black bold) spectra after NO^⋅^ addition. Observed rate constants were 0.10 1/s with Bovine, 2.43 × 10^−3^ 1/s with Horse, and 1.72 × 10^−3^ 1/s with Human metmyoglobin.

To explore how Mb(Fe^+2^-NO) formation varies using Mb from different species, we studied Mb(Fe^+2^-NO) formation in the presence of ascorbate in bovine, equine, and human metmyoglobin. Bovine myoglobin (Fig. 3A) showed the fastest Mb(Fe^+2^-NO) formation, with a rate constant of 0.10/s. In contrast, equine (Fig. 3B) and human (Fig. 3C) metmyoglobin had slower rates, with rate constants of 2.43 × 10^−3^ s^−1^and 1.72 × 10^−3^ s^−1^, respectively. The equilibrium constants for equine myoglobin were (1.5 ± 1.4) × 10^−2^ μM^−1^, and for human metmyoglobin, it was (1.6 ± 1.2) × 10^−2^ μM^−1^. The bimolecular rate constant of ascorbate (*k*_Asc_) for equine metmyoglobin was (1.7 ± 0.9) M^−1^ s^−1^, while for human metmyoglobin, it was (1.1 ± 0.2) M^−1^ s^−1^ (n = 3). In equine and human Mb, NO^•^ addition resulted in only ferric nitrosyl Mb being formed with no reductive nitrosylation, consistent with previous observations at neutral pH [10].

### 3.4. pH dependence

**Fig. 4.**
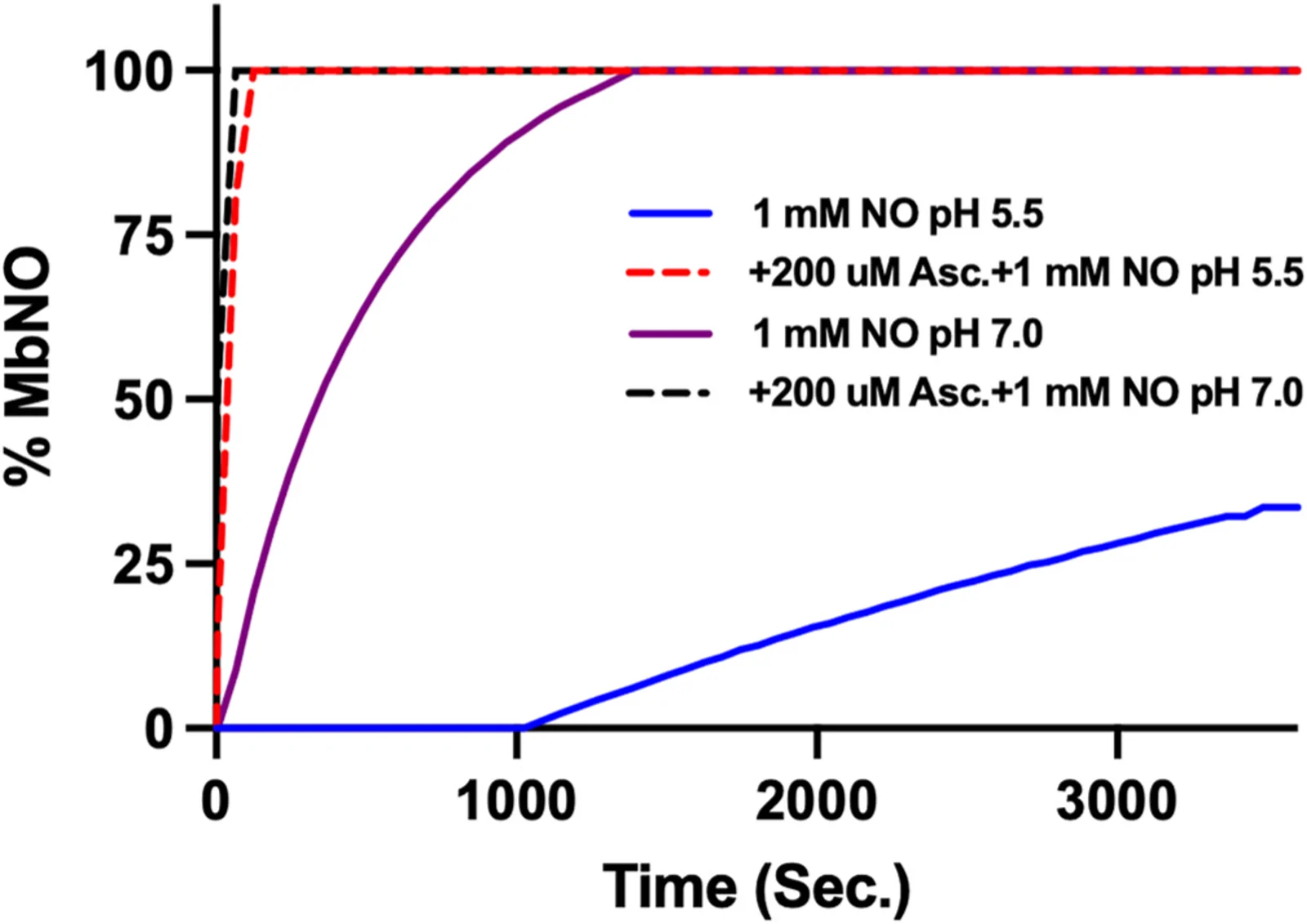
pH dependence. Whereas pH has no effect on the kinetics of Mb(Fe^+2^-NO) formation from 50 μM metmyoglobin and in the presence of ascorbate, it has a large effect when there is no ascorbate.

The classical reductive nitrosylation pathway for the formation of NO-ferroheme depends on hydroxide ion concentration. We investigated the effect of pH on Mb(Fe^+2^-NO) formation to determine if ascorbate simply catalyzes the reaction involving hydroxide ion (Fig. 4). We analyzed the kinetics of this process using 50 μM Metmyoglobin, with and without ascorbate, at 1 mM NO^•^ and at two pH values: 5.5 and 7.0. The formation of Mb(Fe^+2^-NO) in the presence of ascorbate was independent of pH, reaching 100% within less than 2 min of NO^•^ addition. In contrast, the classical reductive nitrosylation reaction required approximately 24 min to achieve complete Mb(Fe^+2^-NO) formation at pH 7, whereas at pH 5.5, this reaction did not fully produce Mb(Fe^+2^-NO) within 1 h. These findings suggest that ascorbate facilitates Mb (Fe^+2^-NO) formation and that this process occurs independently of the classical reductive nitrosylation pathway.

### 3.5. Detection of the ascorbyl radical

**Fig. 5.**
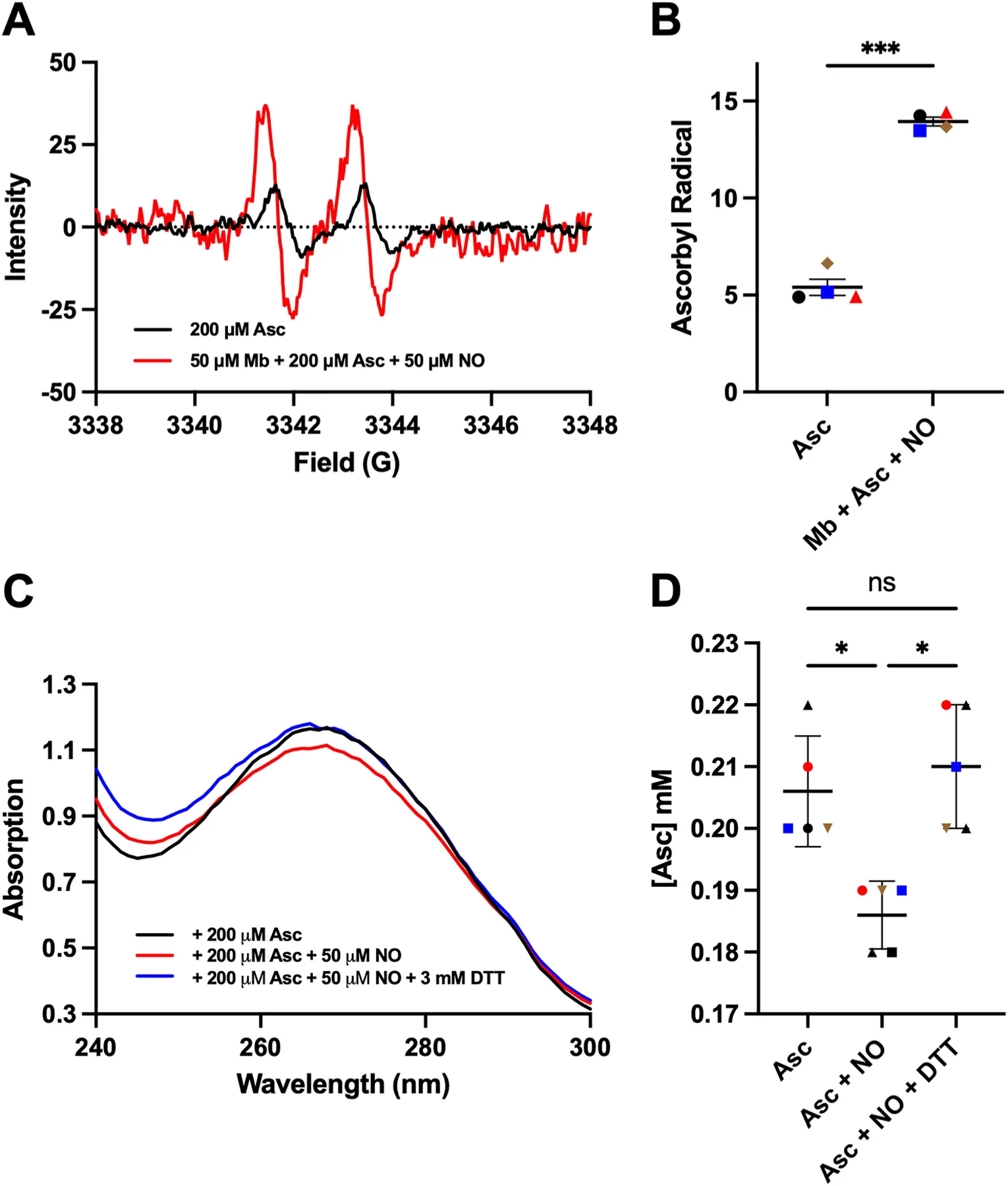
Radical mechanism. (A). EPR. A baseline ascorbyl radical is observed (black line), hyperfine splitting constant = 1.9 G centered at g = 2.0066. Ascorbyl radical growth during the reaction (red signal). (B) Double Integrals of the EPR signals were performed and plotted in arbitrary units for the baseline and reaction mixture (n = 4, P < 0.001). (C) Ascorbate absorption at 265 nm decreases during the reaction forming Mb(Fe+2-NO), but absorbance is restored after adding DTT, consistent with DHA formation from the combination of two ascorbyl radicals. (D) Quantitative analysis of DHA formation was performed using the absorbance at 265 nm of the ascorbate. *P < 0.05, **P < 0.01, One-way Anova measured.

If the mechanism of ascorbate facilitated reductive nitrosylation of myoglobin is similar to the thiol catalyzed reductive nitrosylation (Equation (5) [9]), we should be able to detect an ascorbyl radical by EPR. There is always some ascorbyl radical present in solutions of ascorbate [21] which is what we observed (Fig. 5A, black line). We observed a higher-intensity signal during the reaction of 50 μM metmyoglobin, 200 μM ascorbate, and 50 μM NO^•^ (shown in red), compared to the standard with 200 μM ascorbate alone, supporting the formation of the ascorbyl radical. Performing a double integral of the EPR spectra we observed increased ascorbyl radical to be present after mixing the reactants compared to baseline as summarized in Fig. 5B.

Two ascorbyl radicals combine to form one dehydroascorbate (DHA) and one ascorbate [22]. We examined the formation of DHA by monitoring the ascorbate absorbance at 265 nm (Fig. 5C). The baseline ascorbate absorption when added to metmyoglobin is shown in black. After combining metmyoglobin and 200 μM ascorbate and 50 μM NO^•^, the ascorbate absorption is reduced (red). Adding 3 mM dithiothreitol (DTT), which reduces DHA to ascorbate, increased absorption at 254 nm (blue), consistent with the presence of both the ascorbyl radical and DHA during Mb(Fe^+2^-NO) formation. Quantitative analysis of the absorbance data (Fig. 5D) aligns with the hypothesis that DHA forms and is then converted back to ascorbate upon DTT addition.

### 3.6. Stoichiometry of Mb(Fe^+2^-NO) formation

**Fig. 6.**
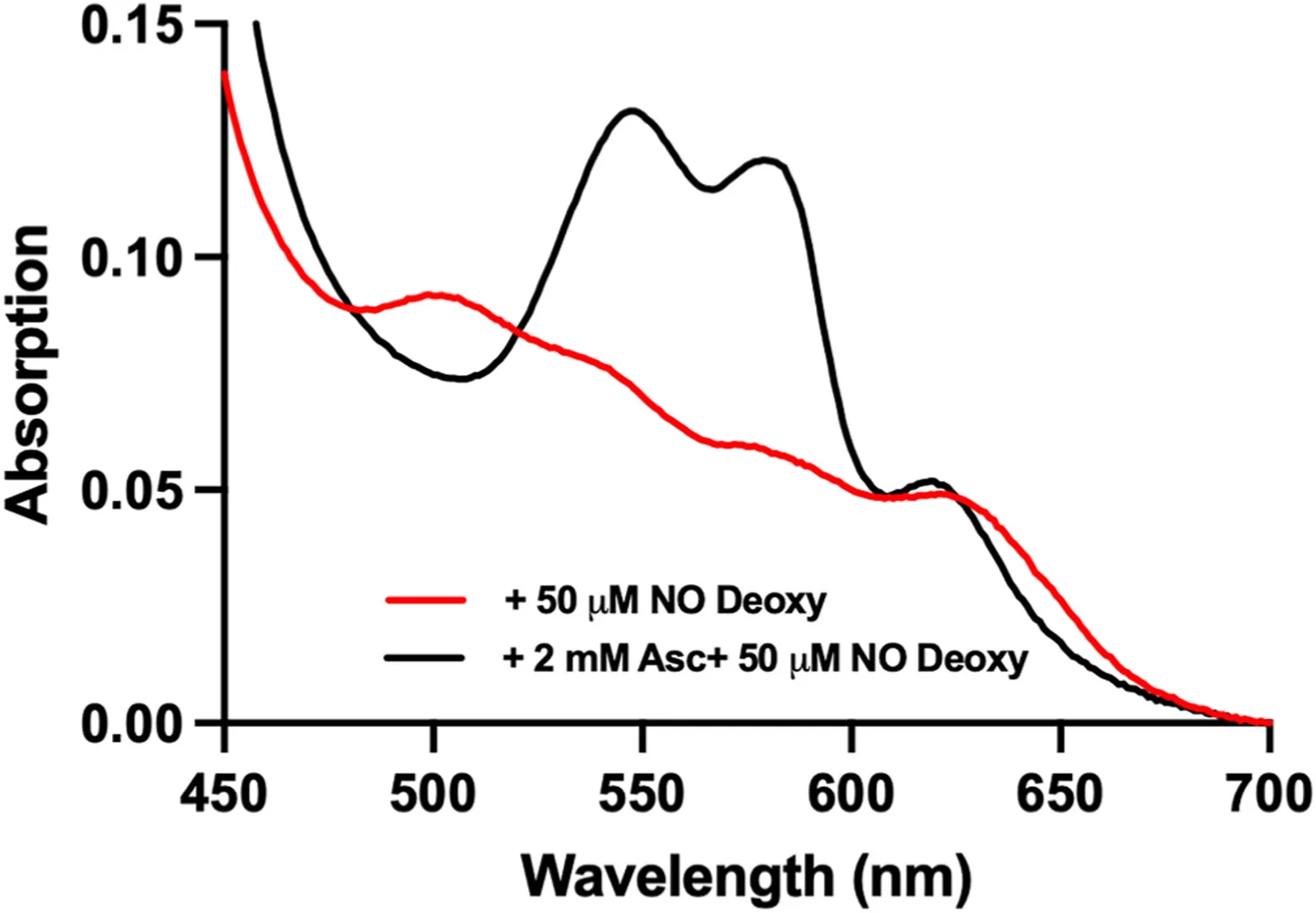
Mb(Fe^+2^-NO) yield from final reaction spectra. Using an extinction of 13.2 mM^−1^cm^−1^ at 545 nm we calculate that 49.4 μM Mb(Fe^+2^-NO) is made from 50 μM metmyoglobin mixed with 50 μM NO^⋅^ with 2 mM ascorbate present, but no Mb(Fe^+2^-NO) is made in the absence of ascorbate.

The classical reductive nitrosylation pathway (Equation (2)) requires two NO^•^ molecules to generate one ferrous nitrosylMb. However, we propose that ascorbate directly reduces the ferric nitrosylMb so that only one NO^•^ molecule is needed to reduce one metMb to one Mb(Fe^+2^-NO). To verify this, we combined equi-molar amounts (50 μM) of metmyoglobin and NO^•^ with ascorbate, yielding Mb(Fe^+2^-NO) in 100% yield (Fig. 6). The average Mb(Fe^+2^-NO) yield from these experiments was (49.8 ± 0.7) μM (n = 3).

Furthermore, since half the ascorbate molecules can be recycled after two ascorbyl radical combine to form one DHA and one ascorbate, one ascorbate molecule can produce two Mb(Fe^+2^-NO) molecules. To test this, we examined Mb(Fe^+2^-NO) formation in the presence of 50 μM metmyoglobin, 25 μM ascorbate, 100 μM NO^•^, and 100 μM (diethylenetriaminepentaacetic acid) DTPA. DTPA served as a chelating agent to stabilize the reaction products and eliminate metal effects. The resultant Mb(Fe^+2^-NO) yield was (85 ± 4)% at pH 7 and (88 ± 4)% at pH 5.5 (n = 3). The yield at the lower pH rules out participation of classical reductive nitrosylation.

### 3.7. Catalysis with myoglobin in molar excess to NO^•^

**Fig. 7.**
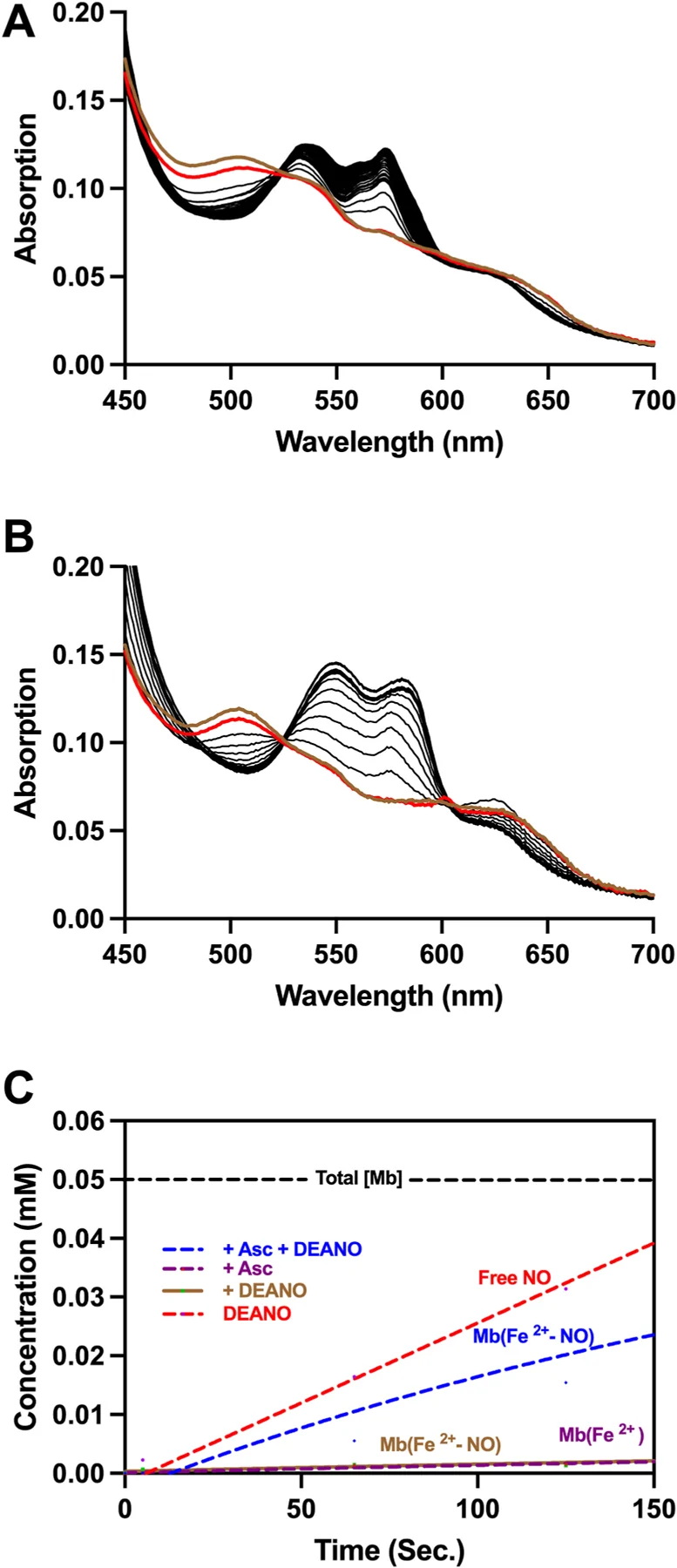
Ascorbate-mediated catalysis with excess Mb to ascorbate. Time-resolved absorption after adding DEA NONOate (250 μM) to metmyoglobin (50 μM) in the absence (A) or presence (B) of 200 μM ascorbate. Brown spectra were taken before adding NO and the red spectra are taken immediately after NONOate addition. Subsequent spectra were collected every minute. (C) Kinetics of Mb species formation after spectral deconvolution resulting from mixing metmyoglobin (50 μM) with DEA NONOate (250 μM), ascorbate (200 μM or both). The total Mb is also shown along with calculated free [NO^•^).

Under physiological conditions, Mb is usually found in molar excess to NO^•^. In order to evaluate whether ascorbate catalyzed reductive nitrosylation occurs under these conditions, we employed a slow NO^•^ donor (DEA NONOate with a half-life of 13 min) and focus on the first several minutes of the reaction. The total NO^•^ released remains below the total Mb concentration (50 μM) for the first 2 min of the reaction. Fig. 7A shows time-resolved absorption spectra of the reaction of NO^•^ and metmyoglobin using the slow NO donor. Time-resolved absorption spectra for the reaction in the presence of 200 μM ascorbate are shown in Fig. 7B. The reaction is clearly much faster in the presence of ascorbate. The spectra in Fig. 7A and B were deconvoluted into basis spectra and we plotted Mb(Fe^+2^-NO) vs time in the presence and absence of ascorbate (Fig. 7C). We also plotted the amount of Mb(Fe^+2^) that is formed over time due to metmyoglobin reduction by ascorbate in the absence of NO^•^. After 2 min the amount of Mb(Fe^+2^-NO) made in the presence of ascorbate and NO^•^ was 16 ± 1 μM while none was made when ascorbate was absent and only 1.4 ± 0.2 μM metmyoglobin was reduced by ascorbate alone within 2 min (n = 3). These data show that ascorbate dramatically catalyzes reductive nitrosylation even when the concentration of metmyoglobin is in excess to NO^•^, and this is not due to direct reduction of the ferric heme followed by NO^•^ binding to the ferrous heme. In Fig. 7C, we also include a plot of the concentration of free NO^•^ (the total NO^•^ minus that bound to ferric and ferrous NO^•^) which remains substantially below the Mb concentration during the time period studied.

### 3.8. Lipid peroxidation

**Fig. 8.**
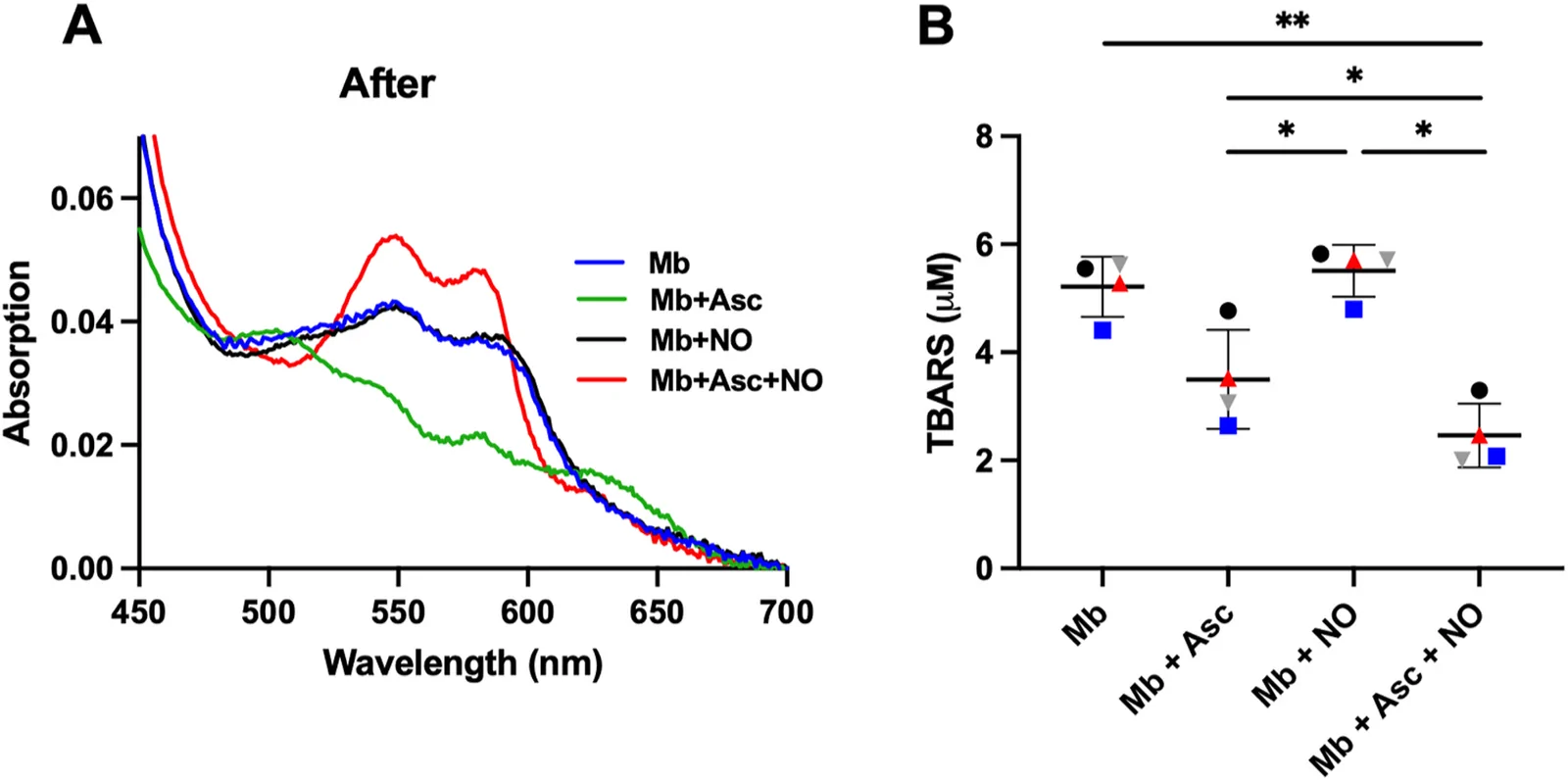
NO-Ferroheme blunts H_2_O_2_ mediated lipid peroxidation. Metmyoglobin (Mb, 50 μM) was treated with 1 mM ascorbate, 100 μM NO^⋅^, neither or both and mixed with 0.4 mM arachidonic acid. Hydrogen peroxide (0.5 mM) was subsequently added. (A) Absorption spectra 10 min after hydrogen peroxide addition. (C) TBARS was measured to assess lipid peroxidation. *P < 0.05, **P < 0.01, One-way Anova measured.

Myoglobin undergoes autoxidation to produce metmyoglobin and ferryl myoglobin, generating free radicals that induce lipid peroxidation. Our hypothesis is that converting metmyoglobin, which can produce oxidizing species by reaction with hydrogen peroxide, into the more chemically inert nitrosylated myoglobin will help reduce lipid peroxidation. To test the hypothesis, metmyoglobin (50 μM) was treated with 1 mM ascorbate, 100 μM NO^•^, neither, or both, and then combined with 0.4 mM arachidonic acid. Hydrogen peroxide (0.5 mM) was added, and the mixture was incubated for 10 min. Absorption spectrometer observations after 10 min of H2O2 treatment indicated that Mb(Fe^+2^-NO) (Fig. 8A, red spectrum) was resistant to H_2_O_2_, demonstrating its stability under these conditions, whereas other treatments resulted in the formation of ferryl and metmyoglobin (Fig. 8A). The TBARS assay used to measure lipid peroxidation (Fig. 8B) shows that ascorbate significantly reduced lipid peroxidation, while the control with NO^•^ alone did not cause notable reduction in lipid peroxidation. The combined treatment of ascorbate and NO, which produced nitrosylated myoglobin, offered greater protection against lipid peroxidation, supporting our hypothesis.

## 4. Discussion

We have shown that glutathione and ascorbate catalyze reductive nitrosylation of metMb under anaerobic conditions. Ascorbate also performed this catalysis under aerobic conditions while glutathione did not (perhaps partially due to competing reactions of NO^•^ with oxygen at the high NO^•^ concentrations employed [23]). The catalysis by ascorbate is demonstrated for bovine, equine, and recombinant human Mb with the reactions being fastest in bovine Mb. With 200 μM NO^•^, addition of 2 mM ascorbate increases the observed rate constant for reductive nitrosylation of bovine Mb 1000-fold. In several conditions shown, NO^•^ addition in the absence of ascorbate does not lead to any ferrous nitrosyl Mb formation whereas when ascorbate is present, ferrous nitrosyl Mb is formed both in aerobic and anaerobic conditions. We also demonstrate that addition of ascorbate and NO^•^ to ferric myoglobin protects against lipid peroxidation by hydrogen peroxide.

Catalysis of reductive nitrosylation of metMb by nitrite has been shown previously [24]. Allosteric effects on reductive nitrosylation kinetics in ferric heme-albumin has also been shown [25,26]. We propose that the mechanism of ascorbate-mediated catalysis of reductive nitrosylation is similar to the thiol catalyzed mechanism involving heme bound albumin or red cell membrane ghosts that we have previously described (Equation (5) [9]) with ascorbate (instead of glutathione) providing an electron to reduce the ferric heme. That ascorbate is not simply catalyzing classical reductive nitrosylation (Equation (2)) is supported by the lack of any significant pH dependence of the kinetics (Fig. 4). Classical reductive nitrosylation depends on the concentration of hydroxide ions so that the kinetics are expected to decrease about 30-fold in going from pH 7 to 5.5 but essentially no effect of pH is observed when ascorbate is present. Moreover, classical reductive nitrosylation requires two NO^•^ molecules to make 1 ferrous nitrosyl Mb, but we see (Fig. 6), that when ascorbate is present only one NO^•^ molecule is needed to make one Mb(Fe^+2^-NO). Rigorous molecular dynamic simulations have elucidated how hydroxyl ion or water coordinates in the heme pocket to reduce the ferric nitrosyl myoglobin and form nitrite [27], it would be interesting to conduct similar simulations to determine how ascorbate reduces the ferric heme and leave a ferrous nitrosyl heme.

The stoichiometry observed in our reaction involving Mb also suggests that the mechanism is not similar to that determined by Kieca et al. using a model thiolate-bound, water soluble heme [28]. Two NO^•^ molecules were required for reductive nitrosylation of the model heme compound whereas our ascorbate catalyzed reaction requires only one. Moreover, Kieca et al. observed that when NO^•^ was mixed in equal amounts to their model heme, no NO-ferroheme was made [28], whereas we observe a nearly 100% yield. The NO-ferroheme yield with stoichiometric and sub-stoichiometric NO^•^:heme that we observed for our heme solubilized in red cell membrane ghosts [9] is also inconsistent with the observed chemistry using the model compound [28]. Comparisons of these systems suggest that the mechanism of NO-ferroheme formation likely depends on the heme coordination and environment. In the case of ascorbate catalyzed reductive nitrosylation of Mb, we propose

(6) $$\text{Mb}\left({\text{Fe}}^{+3}-\text{NO}\right)+\text{Asc}\to \text{Mb}\left({\text{Fe}}^{+2}-\text{NO}\right)+{\text{Asc}}^{•}+{\text{H}}^{+}$$

where Asc refers to ascorbate and Asc^•^ is the relatively stable ascorbyl radical. Subsequently, two ascorbyl radicals can react to form one ascorbate molecule and one dehydroascorbate [22]:

(7) $${\text{Asc}}^{•}+{\text{Asc}}^{•}\to \text{Asc}+\text{DHA}$$

With this regeneration of one ascorbate molecule from two ascorbyl radicals the overall stoichiometry of our reaction is

(8) $$\text{Asc}+2\;\text{Mb}\left({\text{Fe}}^{+3}\right)+2\;{\text{NO}}^{•}\to 2\;\text{Mb}\left({\text{Fe}}^{+2}-\text{NO}\right)+\text{DHA}$$

This stoichiometry is supported by the ferrous nitrosyl yields in Fig. 6 and the associated discussion in the results section. When adding ascorbate to metMb at a molar ratio of 1:2, we saw a yield of 85–88%. Further work is needed to explain why the yield is below the expected 100%.

Examination of the ascorbate absorbance at 265 nm is consistent with DHA formation that is reduced back to ascorbate by addition of dithiothreitol (Fig. 5B). The increase in the abundance of the ascorbyl radical itself (Fig. 5A) also supports our proposed mechanism. In our proposed mechanism for thiol catalyzed reductive nitrosylation in heme-red cell membrane ghosts or heme albumin [9], we did not determine if the reduction occurs through an inner sphere mechanism (where glutathione and NO^•^ are bound to opposite sides of the heme) or an outer sphere mechanism. A recent report focusing on heme-albumin [29] suggests the same inner sphere mechanism that we proposed [9], with glutathione bound distal to NO^•^ binding of the ferric heme. It was also proposed that glutathione binds first (before NO^•^, although this has to be affected by the order of addition) and that some of the bound glutathione reduces the heme forming a thiyl radical bound ferrous heme [29]. For the case of Mb, with the proximal histidine bound to the iron, an outer sphere mechanism is implicated for reduction of Mb (Fe^+3^-NO) by ascorbate.

The concentrations of ascorbate used in this study are within the range of those found under physiological conditions where the abundance of ascorbate varies substantially (tens of micromolar in plasma to 200 μM in skeletal muscle to 10 mM in the brain [30]). Oral or infused ascorbate can increase abundance, for example plasma levels can safely be increased to the millimolar range [30]. On the other hand, steady state levels of NO^•^ are thought to be on the low nanomolar range or below [31]. Higher levels were employed here for most of experiments in order to accurately characterize the chemistry. However, we also conducted experiments using a slow NO^•^ donor (Fig. 7) where the concentration of myoglobin was greater than the concentration of NO^•^ for a substantial part of the reaction and ascorbate-mediated catalysis is clearly observed.

Ascorbate catalyzed reductive nitrosylation could be relevant to meat preservation. Nitric oxide has played a role in meat preservation and NO^•^ formation has been suggested to result from ascorbate reduction of nitrite or reaction with deoxygenated Mb and reductive nitrosylation has also been suggested to play a role [32]. However, ascorbate reduction of nitrite and classical reductive nitrosylation are not likely to be efficient in stored meat which is slightly acidic or at neutral pH. Ascorbate catalyzed reductive nitrosylation could play a role in meat preservation in concert with nitrite-derived NO^•^ by converting ferric Mb to ferrous nitrosyl Mb and thereby reducing lipid peroxidation and Mb degradation.

Ascorbate catalyzed reductive nitrosylation of Mb may also have potential in therapeutics. We showed that NO-ferroheme albumin reduces platelet activation and is more potent at reducing blood pressure in a murine model than NO^•^ itself [9]. In another paper by Lundberg and colleagues that was published simultaneously with ours, it was shown that NO-ferroheme Mb could activate apo-soluble guanylyl cyclase (the primary target for NO^•^ signaling) and lowered blood pressure in rats, suggesting that the NO-ferroheme activates soluble guanylyl cyclase by heme exchange or heme insertion [11]. Thus, one therapeutic strategy is to administer pre-formed NO-ferroheme. In particular, Kleschyov et al. showed that NO-ferroheme Mb confers vasodilation which could be protective in ischemic reperfusion injury and other conditions [11]. Another therapeutic strategy is to administer an NO^•^ source and ascorbate in order to detoxify ferric heme and convert it into a beneficial NO-ferroheme, a process we refer to as heme rehabilitation. A major potential limitation of the heme rehabilitation strategy is that the ascorbate-catalyzed reductive nitrosylation will always compete for NO^•^ with other reactions.

In general, under oxidative stress due to ischemia reperfusion or other conditions, myoglobin can become oxidized to the ferric and ferryl forms and cause lipid peroxidation and other damage [33] which could play a role in some myopathies [34,35]. Lipid peroxidation due to redox cycling of myoglobin has been suggested to play a major role in kidney injury due to rhabdomyolysis [4]. Both NO^•^ (in the absence of super-oxide) [36] and ascorbate [3] are protective against lipid peroxidation when administered separately. Here we have shown that combining the NO and ascorbate, forming NO-ferroheme is more protective than either agent alone in reducing lipid peroxidation from peroxide reactions with ferric Mb. The ferrous nitrosyl Mb formed by treatment with ascorbate and NO^•^ was resistant to hydrogen peroxide treatment as measured by absorption spectroscopy (Fig. 8).

## 5. Conclusion

Ascorbate catalyzes reductive nitrosylation of metMb through an outer sphere reduction of ferric nitrosyl Mb forming NO-ferroheme Mb that has potential in therapeutics through NO^•^ signaling and protection against oxidative stress associated lipid peroxidation.

## Data Availability

Data will be made available on request.
